# Supplementary material for: The Impact of IFN-γ Licensing on Mesenchymal Stromal Cells’ Mediated Immunoregulation and HLA Class II Expression: Emerging Evidence from In Vitro Results
Source: Int J Mol Sci. 2025 Sep 26;26(19):9436. doi: 10.3390/ijms26199436 (PMC12525040; doi:10.3390/ijms26199436)
Supplement: Supplementary file 1 [file ijms-26-09436-s001.zip › ijms-3859695-supplementary.pdf]

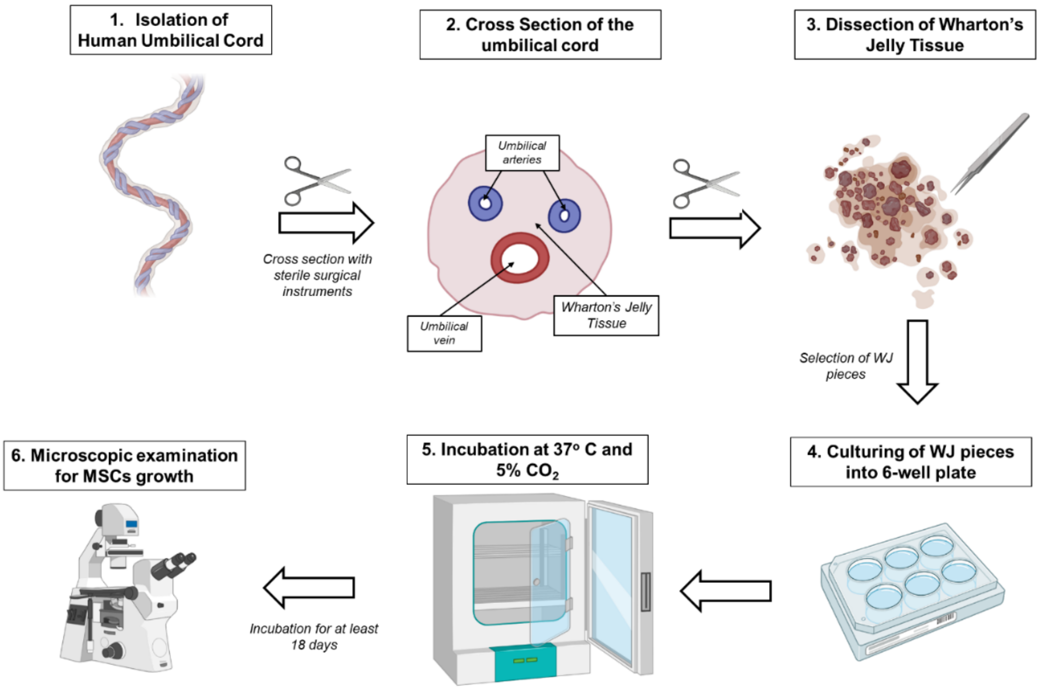

Figure S1. Overview of the experimental procedure.

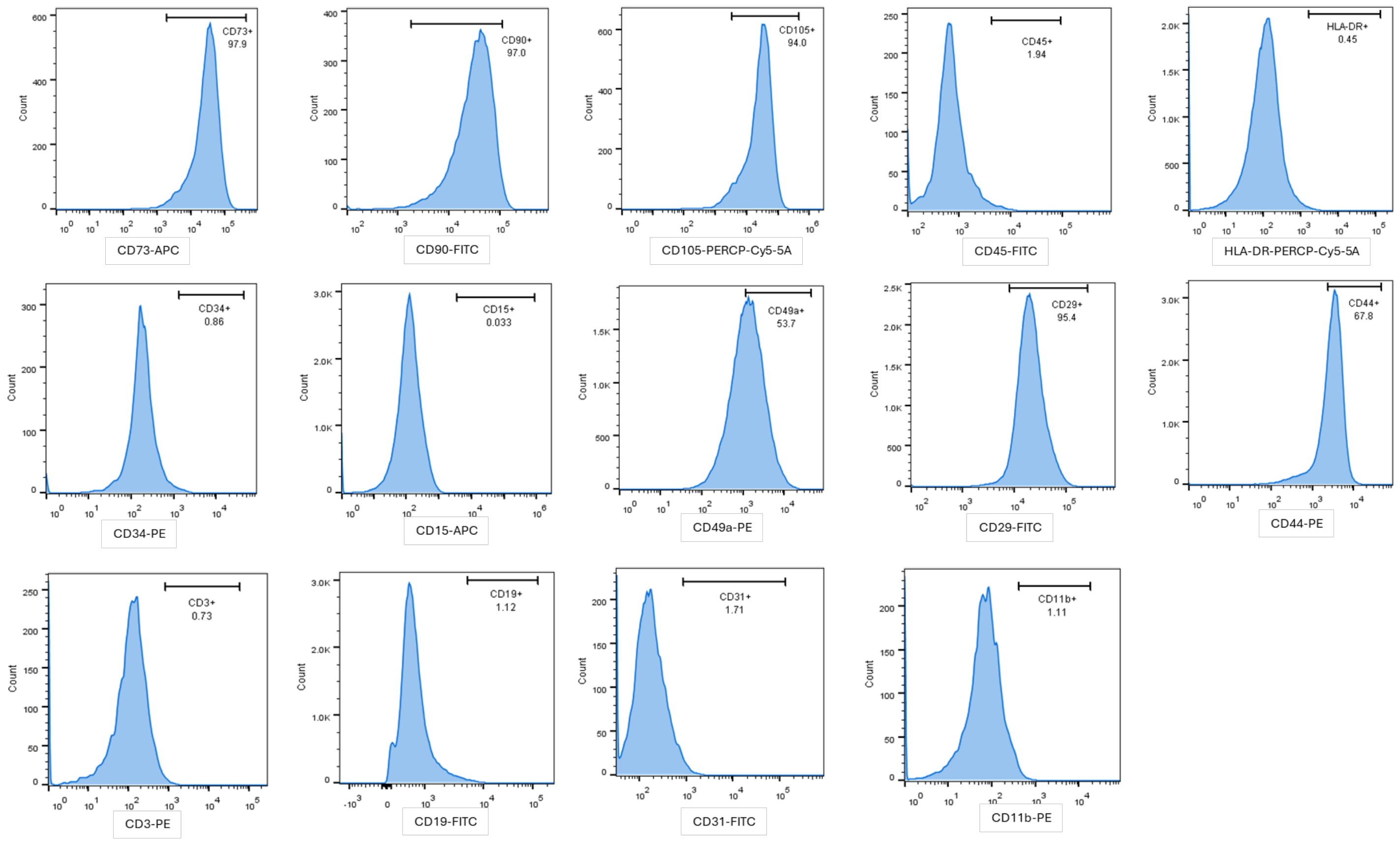

Figure S2. Representative histograms of flow cytometric analysis. WJ-MSCs P4 characterized by high expression (> 90%) for the classical markers CD73, CD90, CD105 and CD29, intermediate expression (> 50%) for the CD49a and CD44 and negative expression for CD45, HLA-DR, CD34, CD15, CD3, CD19, CD31 and CD11b.

Table S1. Percentage of CD markers expression in WJ-MSCs P4.

N=20	**CD73 (%)**	**CD90 (%)**	**CD105 (%)**	**CD45 (%)**	**CD34 (%)**	**HLA-DR (%)**	**CD15 (%)**	**CD49a (%)**	**CD29 (%)**	**CD44 (%)**	**CD3 (%)**	**CD19 (%)**	**CD31 (%)**	**CD11b (%)**
WJ-MSCs	96.7 ± 1.6	96.3 ± 1.9	96 ± 1.6	1 ± 0.5	0.6 ± 0.3	1 ± 0.5	1.2 ± 0.6	63.9 ± 9.8	94.9 ± 1.8	67.4 ± 8.3	0.6 ± 0.2	0.7 ± 0.3	0.5 ± 0.2	0.6 ± 0.3

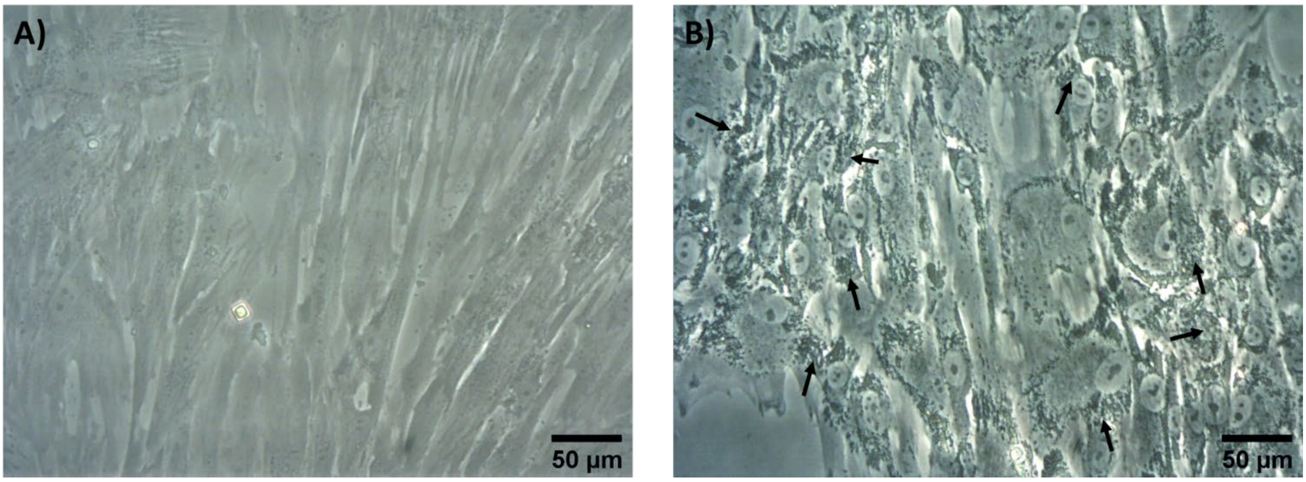

Figure S3. Phenotypic characteristics of WJ-MSCs. Non-primed WJ-MSCs characterized by a low number of intracellular vesicles (A), whereas in IFN-γ primed WJ-MSCs there is clear evidence for the presence of intracellular vesicles (B). Original magnification 20x, and scale bars 50 µm.

Table S2. Percentage of CD markers expression in non-primed and IFN-γ primed WJ-MSCs.

**N=20**	**Non-primed WJ-MSCs** (%)	**IFN-γ primed WJ-MSCs** (%)	***p value***
**CD73**	95 ± 2.1	95.9 ± 2.0	0.197
**CD90**	96 ± 1.9	95.6 ± 1.8	0.445
**CD105**	95.6 ± 1.3	95.1 ± 2.6	0.459
**CD45**	0.9 ± 0.6	0.9 ± 0.5	0.706
**CD34**	0.6 ± 0.4	0.5 ± 0.2	0.564
**CD10**	54.9 ± 13.6	91.4 ± 4.2	**< 0.001**
**CD15**	0.9 ± 0.4	1.1 ± 0.6	0.280
**CD49a**	64.7 ± 7.5	66.7 ± 9.4	0.474
**CD29**	94.9 ± 1.8	94.2 ± 2.4	0.331
**CD44**	66.3 ± 6.6	66.6 ± 7.9	0.909
**CD3**	0.8 ± 0.3	0.6 ± 0.4	0.152
**CD19**	0.6 ± 0.2	0.7 ± 0.3	0.362
**CD31**	0.6 ± 0.2	0.5 ± 0.2	0.136
**CD11b**	0.7 ± 02	0.6 ± 0.2	0.078
**CD80**	0.5 ± 0.2	0.4 ± 0.2	0.249
**CD86**	0.6 ± 0.2	0.7 ± 0.3	0.154
**CD340**	56.8 ± 6.8	53 ± 9.6	0.166
**HLA-DR**	1.1 ± 0.7	63.9 ± 9.8	**< 0.001**
**HLA-DQ**	1.3 ± 0.6	62.1 ± 4.3	**< 0.001**
**HLA-DP**	1.3 ± 0.6	61.4 ± 6.8	**< 0.001**
**7AAD**	5.2 ± 1.1	4.9 ± 1.1	0.320

Table S3. Detailed information regarding the MLR-direct contact.

No	Negative Control	Positive Control	MLR	N-primed WJ-MSCs	IFN-γ Pr WJ-MSCs
1	3100	42500	16050	8590	4561
2	6540	43750	14253	10640	5340
3	5130	38630	15005	12589	6140
4	4750	37450	15185	9960	4762
5	5236	36891	15920	11560	5225
6	5128	40120	17530	12300	4230
7	4956	39856	14420	12450	6165
8	5251	41250	16540	8260	5240
9	5875	40030	14450	11050	6103
10	6780	36432	14560	10390	5080
Average	5275	39691	15391	10779	5285
StDev	968	2274	1030	1451	643

Table S4. Detailed information regarding the MLR-indirect contact.

No	Negative Control	Positive Control	MLR	N-primed WJ-MSCs	IFN-γ Pr WJ-MSCs
1	3100	42500	16050	11230	8150
2	6540	43750	14253	11980	6890
3	5130	38630	15005	12540	9100
4	4750	37450	15185	9480	8560
5	5236	36891	15920	11050	7250
6	5128	40120	17530	12590	6450
7	4956	39856	14420	11350	9120
8	5251	41250	16540	10250	8200
9	5875	40030	14450	9585	6532
10	6780	36432	14560	11780	6890
Average	5275	39691	15391	11184	7714
StDev	968	2274	1030	1058	980

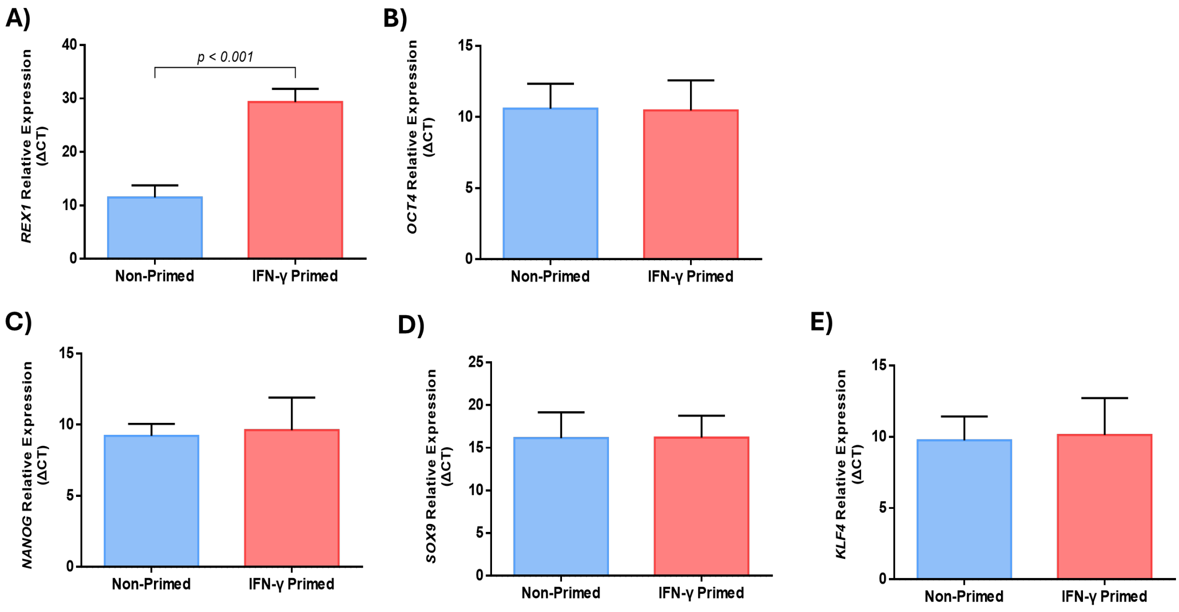

Figure S4. Gene expression level analysis , of the *REX1, OCT4, NANOG, SOX9* and *KLF4*, for non-primed and IFN-γ primed WJ-MSCs, respectively. Specifically, *REX1* (A), *OCT4* (B), *NANOG* (C), *SOX9* (D), and *KLF4* (E) gene expression levels in non primed and IFN-γ primed WJ-MSCs. Statistically significant difference regarding the *REX1* expression level was observed between non -primed and IFN-γ primed WJ-MSCs ($p < 0.001$).

Table S5. Characteristics of IFN-γ primed WJ-MSCs, including secretory profile, immunophenotype and HLA class I and II allele determination using the NGS approach. The presented characteristics used for the clustering of IFN-γ primed WJ-MSCs.

MSCs Sample No	IL-1Ra	IL-10	IL-13	Il-6	TGF-β1	FGF	PDGF	IDO	VEGF	HGF	NO	HLA-DR	CD10	CD340	CD49a	CD73	CD90	CD105	CD45	CD15	CD29	CD44	CD3	CD19	CD31	CD11b	CD34
M0005-07-000249	850	250	210	91	910	1120	1121	1001	1100	985,3	36,9	57,1	95	51	59	98	94,3	98,2	0,51	0,56	92,1	57,1	0,4	1,22	0,85	0,39	0,9
M0005-07-000261	910	290	112	59	1080	1080	985	1610	1025	785,6	41,2	58,7	85	63,2	58,7	95,7	96,7	97,4	1,3	0,87	95,7	65,7	0,12	1,2	0,69	0,39	0,03
M0005-07-000262	587,3	185	200	65	991	1210	1000	1000	1085	689,3	45,1	42	83,2	68,3	42	90,3	91,3	90,3	0,23	1,2	94,8	71,2	0,75	0,42	0,79	0,35	0,1
M0005-07-000264	953	256	120	91	984	984	980	1480	987	1250	56,7	57,1	95,4	57,1	69	97,2	95,2	94,5	0,4	2,3	95	57,1	0,54	0,32	0,87	0,73	1,8
M0005-07-000269	1010	253	132	75	1100	1009	875	1280	1301	1101,1	64,2	58,9	93,6	48	58,9	94,3	98	93,2	0,6	2,7	92,3	58,9	0,56	0,55	0,32	0,64	0,06
M0005-08-004773	635,4	290	129	97	850	840	856	1211	1220	558,3	65,3	56	87,8	35	73	93,7	98,3	96,3	0,3	1	91,3	71,5	0,48	0,3	0,3	0,54	0,05
M0005-07-000290	698	112	92,3	90	902	910	954	958	986	859,6	78,4	56,2	95,6	41	73	95,6	94,6	97,5	0,2	1,7	94	56,2	1	0,24	0,2	0,12	0,03
M0005-07-000291	498,6	154	200	65	654	670	785	786	820	758,3	81,3	65,3	94,5	51	68,3	94,5	97,8	98,3	0,3	1,8	98,5	59,7	2,1	0,36	0,36	0,43	0,02
M0005-07-000297	840	212	155	71	710	701	750	751	750	715,9	53,2	67	92,3	52,3	67	94,5	94,2	95,6	0,7	0,56	90,3	67	0,64	0,58	0,89	0,45	0,56
M0005-07-000518	1200	220	151	69	690	680	1230	1253	1220	1180	38,9	73	97,8	56,7	73	97,8	95,6	97,2	0,8	0,32	92,3	66	0,56	0,36	0,32	0,39	0,23
M0005-07-000547	800	307	101,9	81	805	800	811	812	832	1201,8	60,2	60	89,4	57,8	60	98	98,3	90,2	1	0,34	95,3	63	0,36	0,64	0,54	0,72	0,12
M0005-07-000569	521,3	206	171	53,78	789	911	1320	1450	854	864,3	37,4	63,2	88,2	42	63,2	97,8	96	93,2	2	1,2	93	63	0,45	1,32	0,64	0,56	0,32
M0005-07-000571	910	114	98,9	81	854	915	935	925	920	754,8	69,7	64,3	96,3	40	64,3	96,3	95,2	91,2	0,9	0,85	97,8	62,1	0,48	0,8	0,53	0,91	0,63
M0005-07-000572	1085	185	137	102	786	787	812	915	918	689,2	73,8	84,3	89	39,8	84,3	95,4	93,4	93,2	0,8	1,8	98,3	81,2	0,56	0,7	0,72	0,56	0,62
M0005-07-000584	1050	212	140	120	815	815	854	1420	1350	810,9	79,8	87,1	96,7	62,1	87,1	96,7	93,5	99,5	0,9	0,56	95,6	87,1	0,78	1	0,56	0,32	1
M0005-07-000590	720	256	89,3	130	1150	942	902	954	965	990,7	59,8	62,1	85,2	53	62,1	97,8	95,6	96,3	1,3	0,77	90,8	62,1	0,42	1,2	0,32	0,56	0,07
M0005-07-000596	920	289	185	95	785	758	782	789	815	815,3	87,8	67	94,2	54	69	94,8	97,3	96,8	0,4	0,78	92,02	67	0,63	0,7	0,56	0,89	0,09
M0005-08-004886	610,2	312	190	82,9	945	945	930	1120	1078	951,6	79,72	70	88,9	55	70	98,2	96,3	96	0,8	0,62	93,2	72	0,54	0,9	0,911	0,54	0,36
M0005-08-004874	603	340	197	89	810	815	878	858	915	745,7	75,3	69	89,2	61	69	97,5	95,8	94,3	1,2	1	97	71,2	0,4	1	0,32	1,2	1,3
M0005-08-001088	810	309	182	105	854	856	1220	1321	1378	891,2	80,1	59	91,2	71	63	93,2	94,1	93,4	2,2	1,3	95,1	73	0,05	0,56	0,21	0,62	1

MSCs Sample No	HLA-A		HLA-B		HLA-C		HLA-DRB1		HLA-DQB1		HLA-DPB1	
M0005-07-000249	HLA-A*11:01:01	HLA-A*24:02:01	HLA-B*35:01:01	HLA-B*51:01:01	HLA-C*01:02:01	HLA-C*04:01:01	HLA-DRB1*11:01:01	HLA-DRB1*11:01:01	HLA-DQB1*03:01:01	HLA-DQB1*03:01:01	HLA-DPB1*02:01:02	HLA-DPB1*04:01:01
M0005-07-000261	HLA-A*02:01:01	HLA-A*11:01:01	HLA-B*07:02:01	HLA-B*51:01:01	HLA-C*01:02:01	HLA-C*07:02:01	HLA-DRB1*14:01:01	HLA-DRB1*15:01:01	HLA-DQB1*05:03:01	HLA-DQB1*06:02:01	HLA-DPB1*04:01:01	HLA-DPB1*15:01:01
M0005-07-000262	HLA-A*26:01:01	HLA-A*33:01:01	HLA-B*14:02:01	HLA-B*18:01:01	HLA-C*08:02:01	HLA-C*12:03:01	HLA-DRB1*01:02:01	HLA-DRB1*11:04:01	HLA-DQB1*03:01:01	HLA-DQB1*05:01:01	HLA-DPB1*04:01:01	HLA-DPB1*04:01:01
M0005-07-000264	HLA-A*03:01:01	HLA-A*24:02:01	HLA-B*51:01:01	HLA-B*52:01:01	HLA-C*12:02:02	HLA-C*12:03:01	HLA-DRB1*11:04:01	HLA-DRB1*15:02:01	HLA-DQB1*03:01:01	HLA-DQB1*03:01:01	HLA-DPB1*04:02:01	HLA-DPB1*23:01:01
M0005-07-000269	HLA-A*01:01:01	HLA-A*23:01:01	HLA-B*07:02:01	HLA-B*44:03:01	HLA-C*04:01:01	HLA-C*07:02:01	HLA-DRB1*07:01:01	HLA-DRB1*14:54:01	HLA-DQB1*02:02:01	HLA-DQB1*05:03:01	HLA-DPB1*04:01:01	HLA-DPB1*04:02:01
M0005-08-004773	HLA-A*02:01:01	HLA-A*26:01:01	HLA-B*40:01:02	HLA-B*51:01:01	HLA-C*03:04:01	HLA-C*14:02:01	HLA-DRB1*14:54:01	HLA-DRB1*16:01:01	HLA-DQB1*05:02:01	HLA-DQB1*05:03:01	HLA-DPB1*04:01:01	HLA-DPB1*05:01:01
M0005-07-000290	HLA-A*26:01:01	HLA-A*68:02:01	HLA-B*39:01:01	HLA-B*53:01:01	HLA-C*04:01:01	HLA-C*12:03:01	HLA-DRB1*13:02:01	HLA-DRB1*16:02:01	HLA-DQB1*05:02:01	HLA-DQB1*06:09:01	HLA-DPB1*03:01:01	HLA-DPB1*04:02:01
M0005-07-000291	HLA-A*01:01:01	HLA-A*23:01:01	HLA-B*49:01:01	HLA-B*57:01:01	HLA-C*06:02:01	HLA-C*07:01:01	HLA-DRB1*11:01:01	HLA-DRB1*13:01:01	HLA-DQB1*03:01:01	HLA-DQB1*06:03:01	HLA-DPB1*03:01:01	HLA-DPB1*04:01:01
M0005-07-000297	HLA-A*01:01:01	HLA-A*24:02:01	HLA-B*35:03:01	HLA-B*37:01:01	HLA-C*04:01:01	HLA-C*06:02:01	HLA-DRB1*04:01:01	HLA-DRB1*12:01:01	HLA-DQB1*03:01:01	HLA-DQB1*03:01:01	HLA-DPB1*04:01:01	HLA-DPB1*04:01:01
M0005-07-000518	HLA-A*02:01:01	HLA-A*24:02:01	HLA-B*18:01:01	HLA-B*35:03:01	HLA-C*07:01:01	HLA-C*12:03:01	HLA-DRB1*11:04:01	HLA-DRB1*14:54:01	HLA-DQB1*03:01:01	HLA-DQB1*05:03:01	HLA-DPB1*04:01:01	HLA-DPB1*04:02:01
M0005-07-000547	HLA-A*11:01:01	HLA-A*24:02:01	HLA-B*35:01:01	HLA-B*35:02:01	HLA-C*04:01:01	HLA-C*04:01:01	HLA-DRB1*11:04:01	HLA-DRB1*14:54:01	HLA-DQB1*03:01:01	HLA-DQB1*05:03:01	HLA-DPB1*02:01:02	HLA-DPB1*104:01:01
M0005-07-000569	HLA-A*01:01:01	HLA-A*24:02:01	HLA-B*35:02:01	HLA-B*37:01:01	HLA-C*04:01:01	HLA-C*06:02:01	HLA-DRB1*11:04:01	HLA-DRB1*15:02:01	HLA-DQB1*03:01:01	HLA-DQB1*06:01:01	HLA-DPB1*02:01:02	HLA-DPB1*04:01:01
M0005-07-000571	HLA-A*24:02:01	HLA-A*33:01:01	HLA-B*18:01:01	HLA-B*35:03:01	HLA-C*05:01:01	HLA-C*12:03:01	HLA-DRB1*03:01:01	HLA-DRB1*11:04:01	HLA-DQB1*03:01:01	HLA-DQB1*05:01:01	HLA-DPB1*04:01:01	HLA-DPB1*04:02:01
M0005-07-000572	HLA-A*02:01:01	HLA-A*03:01:01	HLA-B*38:01:01	HLA-B*51:01:01	HLA-C*12:03:01	HLA-C*15:02:01	HLA-DRB1*13:01:01	HLA-DRB1*13:01:01	HLA-DQB1*06:03:01	HLA-DQB1*06:03:01	HLA-DPB1*03:01:01	HLA-DPB1*04:01:01
M0005-07-000584	HLA-A*02:01:01	HLA-A*03:01:01	HLA-B*44:05:01	HLA-B*51:01:01	HLA-C*02:02:02	HLA-C*15:02:01	HLA-DRB1*11:01:01	HLA-DRB1*14:01:01	HLA-DQB1*03:01:01	HLA-DQB1*05:03:01	HLA-DPB1*04:01:01	HLA-DPB1*10:01:01
M0005-07-000590	HLA-A*02:01:01	HLA-A*03:01:01	HLA-B*35:01:01	HLA-B*44:02:01	HLA-C*04:01:01	HLA-C*04:01:01	HLA-DRB1*01:01:01	HLA-DRB1*15:01:01	HLA-DQB1*05:01:01	HLA-DQB1*06:02:01	HLA-DPB1*04:01:01	HLA-DPB1*14:01:01
M0005-07-000596	HLA-A*02:01:01	HLA-A*03:01:01	HLA-B*18:05:01	HLA-B*44:02:01	HLA-C*05:01:01	HLA-C*12:03:01	HLA-DRB1*12:10:01	HLA-DRB1*16:01:01	HLA-DQB1*03:01:01	HLA-DQB1*05:02:01	HLA-DPB1*04:01:01	HLA-DPB1*06:01:01
M0005-08-004886	HLA-A*02:01:01	HLA-A*32:01:01	HLA-B*35:03:01	HLA-B*44:02:01	HLA-C*04:01:01	HLA-C*05:01:01	HLA-DRB1*01:01:01	HLA-DRB1*03:01:01	HLA-DQB1*02:01:01	HLA-DQB1*05:01:01	HLA-DPB1*04:01:01	HLA-DPB1*04:01:01
M0005-08-004874	HLA-A*02:01:01	HLA-A*32:01:01	HLA-B*39:31:01	HLA-B*51:01:01	HLA-C*12:03:01	HLA-C*15:02:01	HLA-DRB1*11:01:01	HLA-DRB1*12:10:01	HLA-DQB1*03:01:01	HLA-DQB1*03:01:01	HLA-DPB1*02:01:02	HLA-DPB1*04:02:01
M0005-08-001088	HLA-A*01:01:01	HLA-A*03:01:01	HLA-B*35:01:01	HLA-B*52:01:01	HLA-C*04:01:01	HLA-C*12:02:02	HLA-DRB1*01:01:01	HLA-DRB1*15:02:01	HLA-DQB1*05:01:01	HLA-DQB1*06:01:01	HLA-DPB1*04:01:01	HLA-DPB1*13:01:01

A)

B)

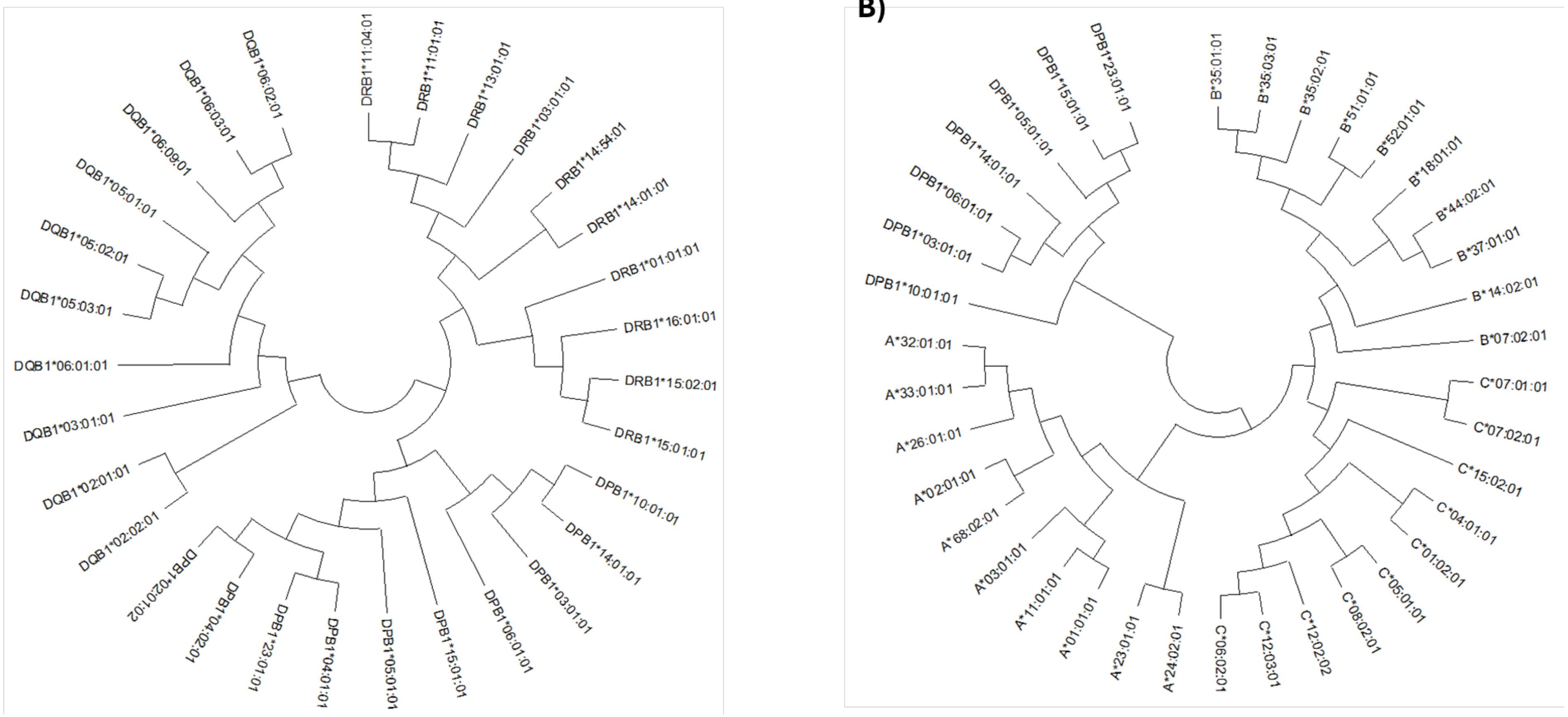

Figure S5. Neighbor joining tree of the most frequent HLA class I (A) and class II (B) alleles.

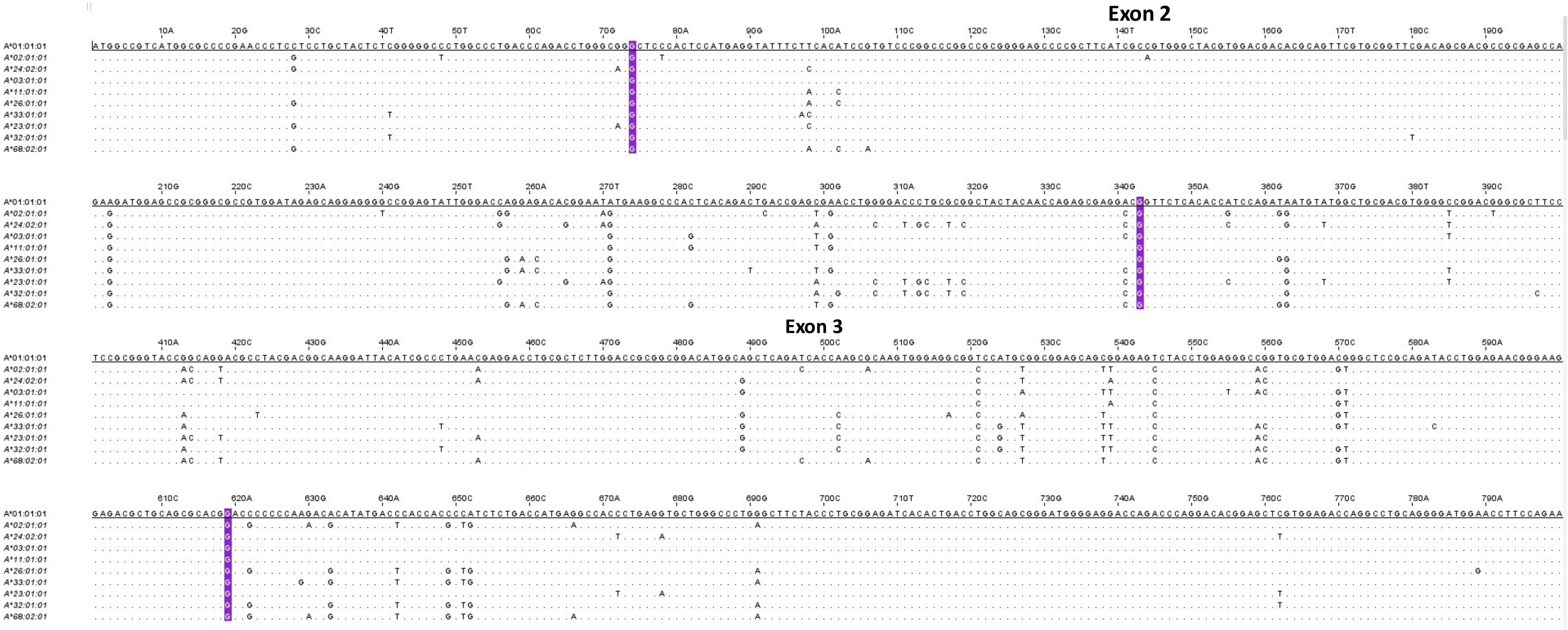

Figure S6. Nucleotide sequence of HLA-A alleles.

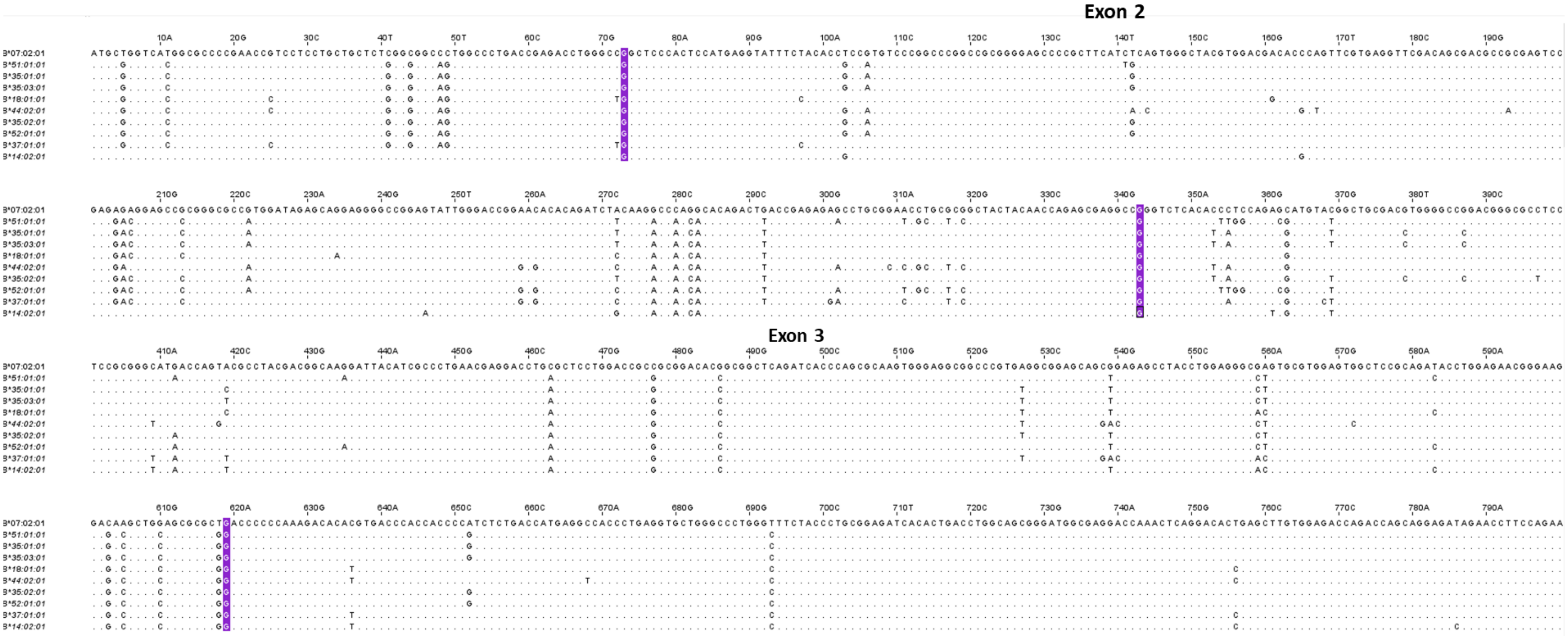

Figure S7. Nucleotide sequence of HLA-B alleles.

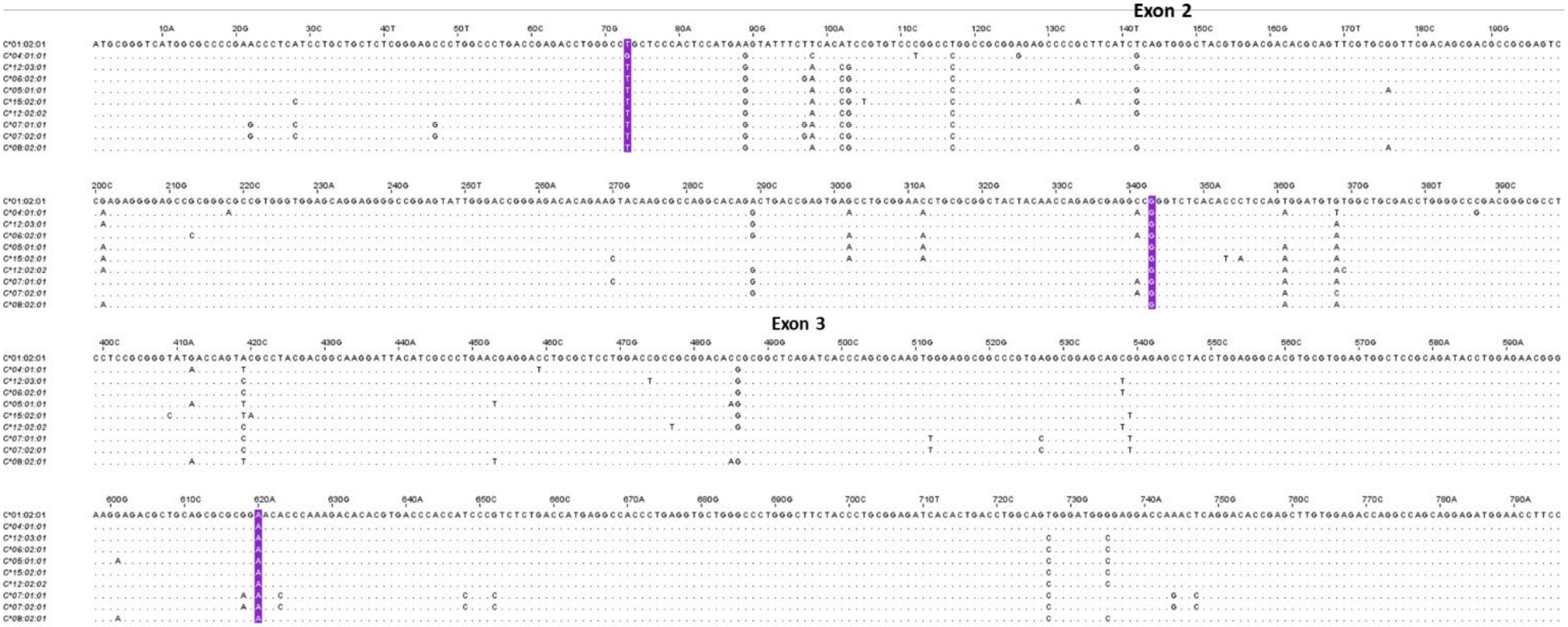

Figure S8. Nucleotide sequence of HLA-C alleles.

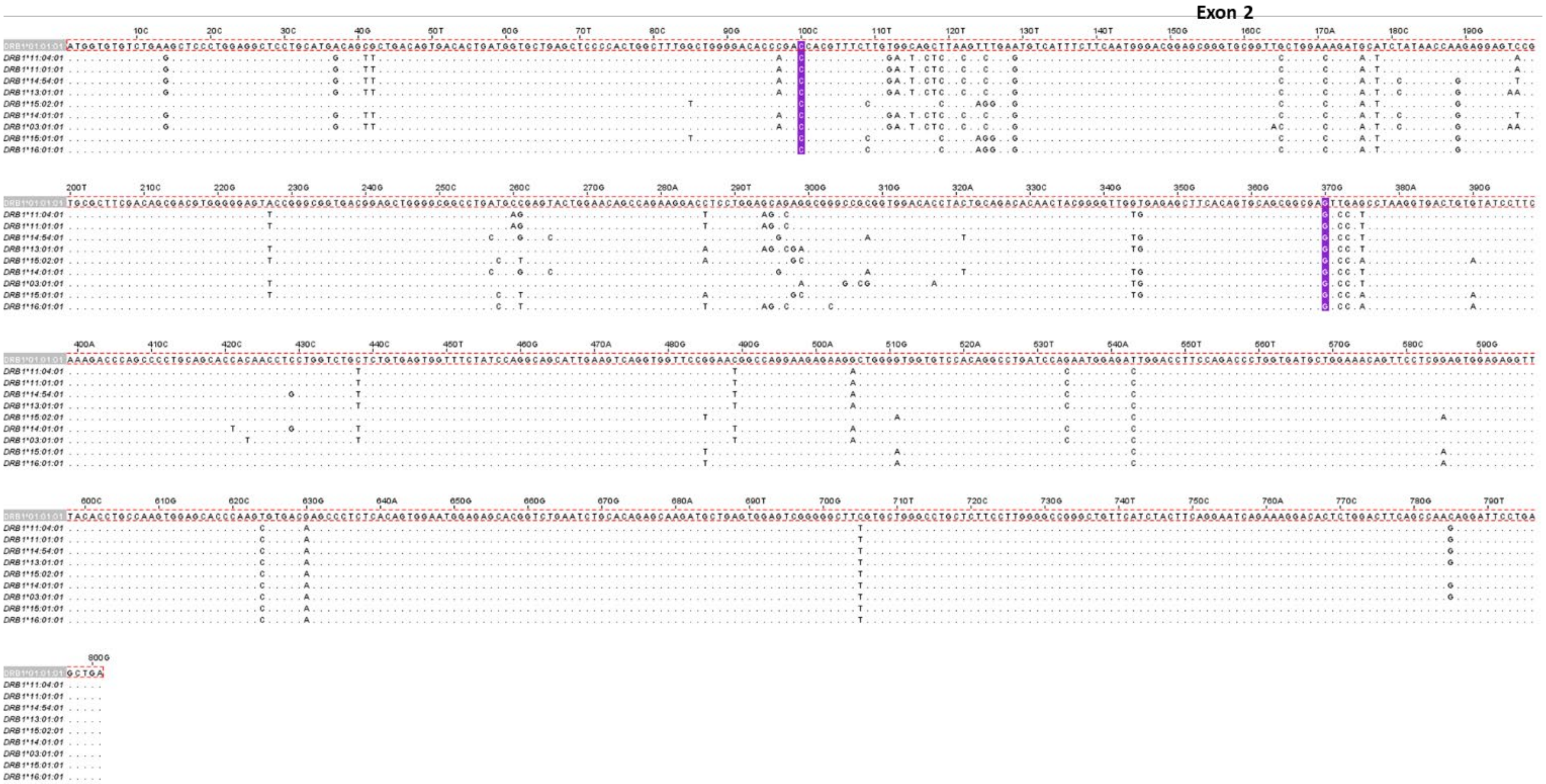

Figure S9. Nucleotide sequence of HLA-DRB1 alleles.

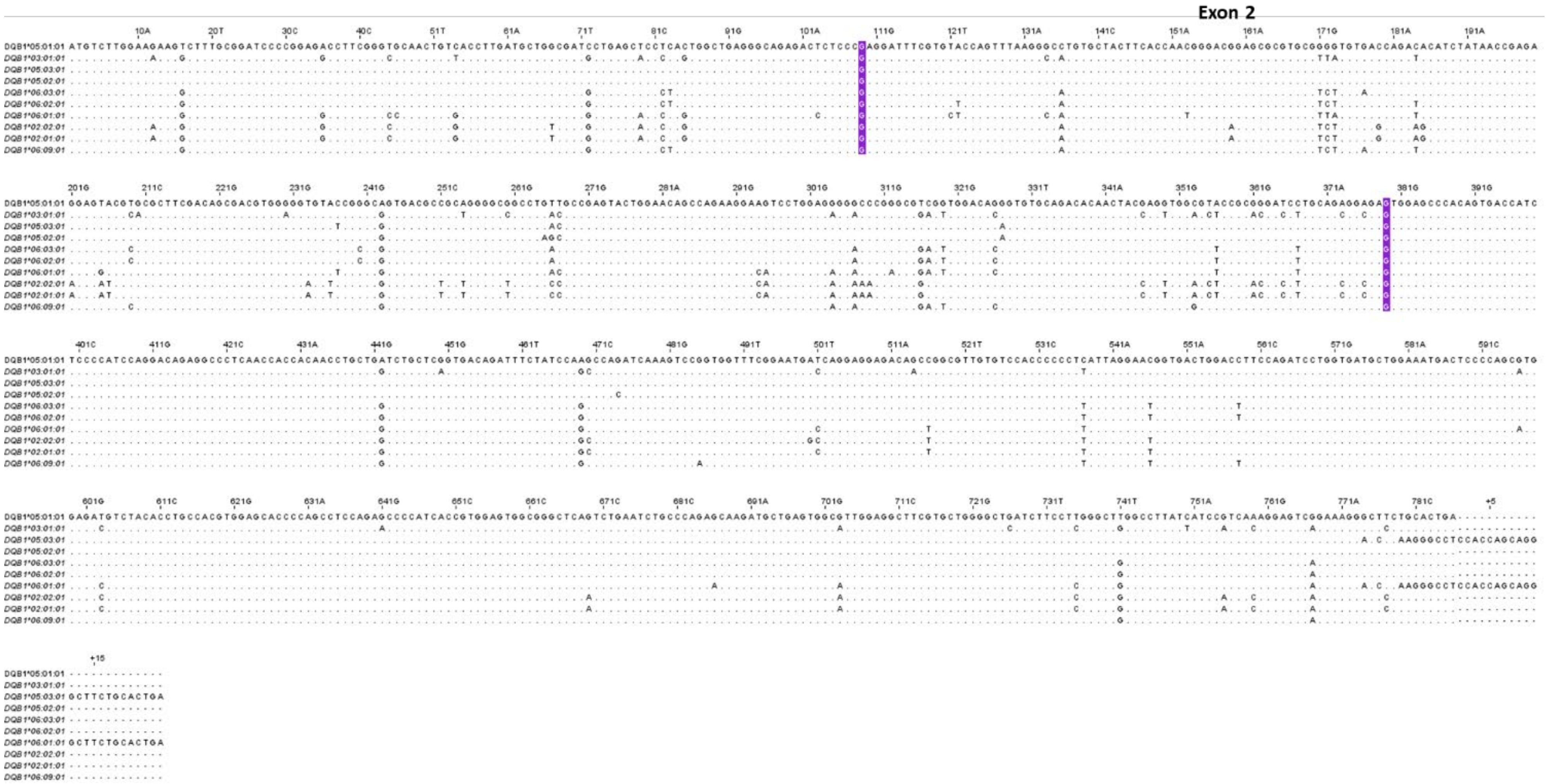

Figure S10. Nucleotide sequence of HLA-DQB1 alleles.

Exon 2

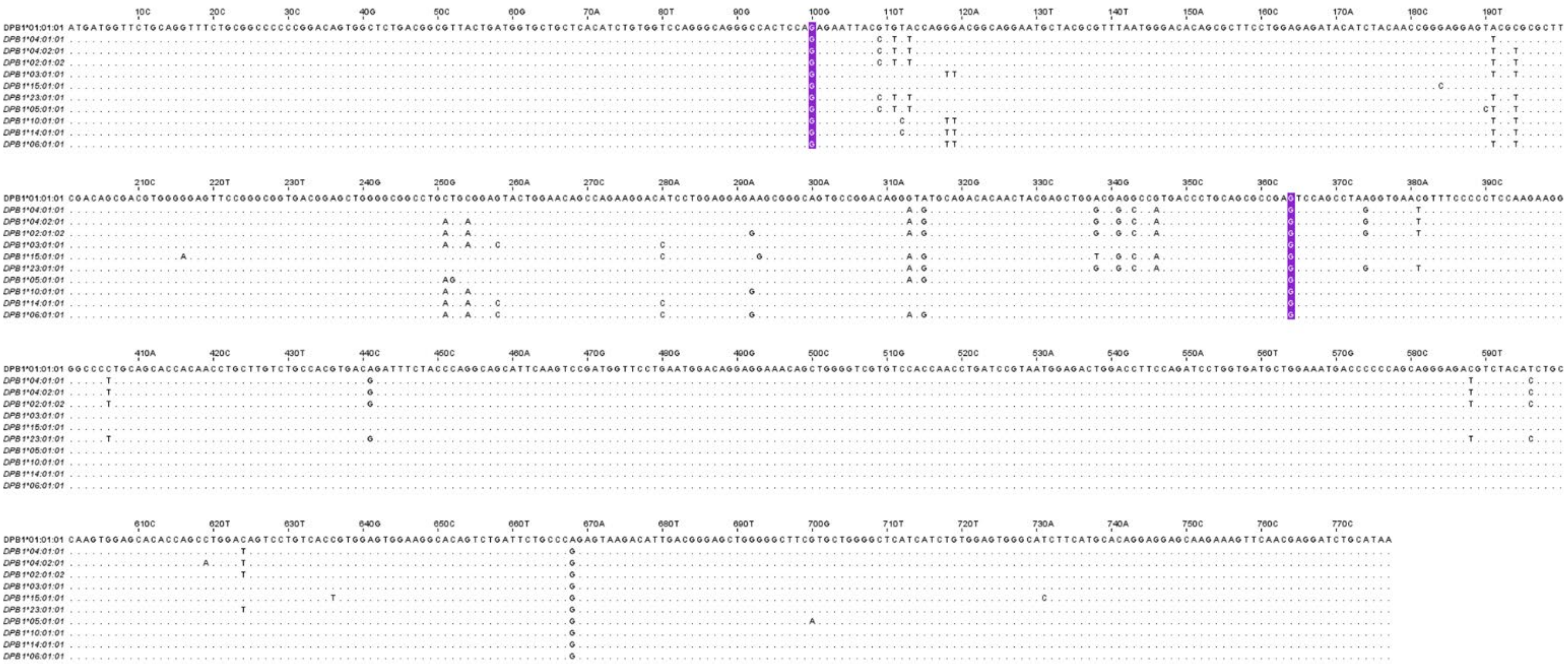

Figure S11. Nucleotide sequence of HLA-DPB1 alleles.
